# Deciphering the pathogen heterogeneity for precise diagnosis and personalized therapeutics of infections after kidney transplantation: insights from metagenomic next-generation sequencing

**DOI:** 10.3389/fcimb.2024.1456407

**Published:** 2024-11-14

**Authors:** Xin Ye, Yuxin Lin, Jiangnan Yang, Baocui Qi, Xuedong Wei, Yuhua Huang, Liangliang Wang

**Affiliations:** ^1^ Department of Urology, The First Affiliated Hospital of Soochow University, Suzhou, China; ^2^ Department of Medicine, Dinfectome Inc., Nanjing, China

**Keywords:** kidney transplantation, metagenomic next-generation sequencing, pathogen detection, clinical decision-making, infection

## Abstract

**Introduction:**

The aim of this study was to compare the detection performance of mNGS against that of conventional tests (CT) in patients suffering from infection after kidney transplantation.

**Methods:**

A total of 138 samples from 85 kidney transplant patients with acute or chronic infections were simultaneously analyzed using mNGS and CT from July 2021 to August 2023.

**Results:**

Compared with CT, mNGS demonstrated a higher sensitivity (95.96% vs. 27.27%) but lower specificity (48.72% vs. 84.62%) in pathogen detection. Moreover, mNGS exhibited significant advantages in detecting mixed and rare infections. The pathogens commonly identified in kidney transplant patients were severe acute respiratory syndrome coronavirus 2 (SARS-CoV-2), followed by Pneumocystis jirovecii and Cytomegalovirus (CMV). mNGS guided the precise clinical diagnosis in 89.13% of cases and assisted in altering therapeutics from empirical antibiotic approaches to personalized plans in 56.10% of cases, including treatment escalation (40.65%), initiation (11.38%), drug adjustment (3.25%), and de-escalation (0.81%).

**Discussion:**

Our study demonstrated the superior detection performance of mNGS and its significant clinical value. This reflected the great potential of mNGS as a complementary clinical detection technology for kidney transplant patients.

## 1 Introduction

Kidney transplantation is considered the most effective treatment for end-stage kidney disease (Lentine et al., 2022). However, extensive and long-term use of immunosuppressants in transplant recipients significantly weakens their immune function and increases susceptibility to infections. Compared to the general population, transplant recipients are at a higher risk of developing complicated infections, such as opportunistic, mixed, rare bacterial infections, and severe infections (Sayegh and Carpenter, 2004; Fishman, 2007; Augustine, 2018; Agrawal et al., 2022). These infections lead to delayed diagnosis, untimely treatment and poor prognosis (Abbott et al., 2002; Fishman, 2017; Brune et al., 2022; Bouwmans et al., 2024). Therefore, a rapid and accurate diagnostic method for infections in kidney transplant recipients is crucial.

Among current diagnostic methods, such as microbiological cultures, serology, antigen and antibody tests, cultures are widely regarded as the gold standard. However, these methods often come with drawbacks, including long turnaround time (TAT), low positive rates, and a frequent failure to detect many pathogens early in the course of infection (Schuele et al., 2021; Jenks et al., 2023). These challenges are pronounced in kidney transplant recipients, making timely and accurate pathogen detection critical.

In recent years, metagenomic next-generation sequencing (mNGS) has emerged as an unbiased method capable of detecting all infectious pathogens without the need for prior clinical prediction (Duan et al., 2022; Tian et al., 2022). mNGS overcomes many disadvantages associated with conventional tests (CT), broadening its clinical applications significantly, including in solid organ transplantation (Schuele et al., 2021; Zhang et al., 2021; Lv et al., 2024; Xing et al., 2024). Despite its promising capabilities, there is a lack of comprehensive and large-scale research in renal transplantation patients suffering from infections. Most past studies focused on the diagnostic advantages of mNGS for specific pathogens, often neglecting its unbiased nature (Zhang et al., 2021; Duan et al., 2022; Tian et al., 2022; Xing et al., 2024). This narrow scope, along with limited sample sizes and a lack of large-scale validation, restricts the full potential of mNGS in kidney transplant recipients.

This study aimed to compare the detection performance of mNGS and CT across different sample types, and to assess the clinical value of mNGS in the diagnosis and treatment of kidney transplant recipients.

## 2 Materials and methods

### 2.1 Study patients

The study retrospectively reviewed the medical records of 85 patients who underwent kidney transplantation at The First Affiliated Hospital of Soochow University in Suzhou, China, between July 2021 and August 2023. These patients were suspected of having lung infection with respiratory symptoms (e.g., cough, sputum, chest tightness, and dyspnea), suspected urinary tract infections (e.g., frequent urination, urgency, and pain), or unexplained fever. Samples were collected from the primary lesion site, as initially diagnosed by the clinician. Each sample was regularly subjected to paired testing, comparing conventional microbiological detection methods and mNGS. This study was approved by the Ethics Committee of the First Affiliated Hospital of Soochow University (approval number: 2023-181) and was conducted according to the principles of the Helsinki Declaration. The Ethics Committee waived the need for informed consent.

### 2.2 Inclusion and exclusion criteria

The inclusion criteria for this study were as follows: (1) kidney transplant recipients aged 18 to 65 years old; (2) patients with suspected infection; (3) patients agreed to mNGS for inspection; and (4) samples analyzed using both CT and mNGS. The exclusion criteria were: (1) unobtainable samples; (2) patients under 18 years old; (3) HIV infection; and (4) unqualified specimens or incomplete clinical data.

### 2.3 Conventional microbiological tests

Traditional etiological examination for suspected infection were retrospectively analyzed. Conventional tests included bacterial and fungal cultures, galactomannan testing, Cytomegalovirus (CMV) PCR, COVID-19 PCR. Clinical assessments of infection or non-infection, as well as identification of pathogenic agents, were based on the patient’s symptoms, chest x-ray or computed tomography scan, smear and culture, and mNGS results. This assessment was conducted by two experienced specialists, with discrepancies resolved by consulting a third expert. Pathogens detected by CTs or mNGS were considered true positives only if they were consistent with the final clinical diagnosis; otherwise, they were considered false positives.

### 2.4 Sample collection

Blood samples (8–10 mL per sample) were collected from the cubital vein using a DNA/RNA negative pressure anticoagulant collection tube, followed by gently inverting the tube 3–4 times to ensure proper mixing. At least 2 mL of sputum sample was collected from each patient by coughing forcefully to expel deep respiratory secretions after rinsing the mouth with clean water 2–3 times. The ideal specimen was the first sputum of the morning. For bronchoalveolar lavage fluid (BALF) collection, local anesthesia was applied to the patient’s throat, followed by introducing of a fiberoptic bronchoscope. The affected pulmonary segment or subsegment was lavaged with sterile saline, either at 37°C or room temperature. Next, 20–60 mL of saline was administered multiple times (typically 4–5 times) until a total of 100–300 mL was lavaged. The fluid was then recovered by aspiration, and 3–50 mL of the recovered fluid was collected in a sterile collection tube. For urine samples, at least 3 mL of midstream urine sample was collected from each patient after properly cleaning or disinfecting the urethral opening. All samples were collected with strict adherence to sterility principles and stored in sterile collection tubes sealed with sealing film. Once collected, samples were delivered to the laboratory for testing within 24 hours and transported at 4°C.

### 2.5 Nucleic acid extraction

All samples were collected from patients according to standard procedures. DNA was extracted using the TIANamp Micro DNA Kit (Tiangen Biotech, Beijing, China) according to the manufacturer’s protocols. The quantity and quality of DNA were assessed using Qubit (Thermo Fisher Scientific) and NanoDrop (Thermo Fisher Scientific) instruments, respectively. Nuclease-free water was used as a negative control (NTC) and processed in parallel with the clinical samples throughout the entire mNGS procedure.

### 2.6 Library preparation and sequencing

DNA libraries were prepared using the Hieff NGS C130P2 OnePot II DNA Library Prep Kit for MGI (Yeasen Biotechnology) according to the manufacturer’s protocols. DNA libraries were qualified using the Agilent 2100, and 50 bp single-end sequencing was performed on the MGISEQ-200 platform (MGI Tech, China). Approximately 20 million reads were generated per sample.

### 2.7 Bioinformatics analysis

We used an in-house developed bioinformatics pipeline for microorganism identification, as previously described (Lv et al., 2024; Song et al., 2024). Raw sequencing data were processed using bcl2fastq2 (version 2.20) to split the data. High-quality sequencing data were obtained following the removal of low-quality reads, adapter contamination, and duplicates, and short (read length <36 bp) reads using Trimmomatic (version 0.36). Human host sequences were excluded by mapping to the human reference genome (hs37d5) using bowtie2 software. The remaining data were aligned with the NCBI microorganism genome database for microorganism identification using Kraken2(version 2.0.7) and for species abundance estimation using Bracken (version 2.5.0). The microorganism genome database contained genomes or scaffolds of bacteria, fungi, viruses and parasites (download from GenBank release 238, ftp://ftp.ncbi.nlm.nih.gov/genomes/genbank/). Consequently, the microbial composition of the samples was determined. Pathogens that met the threshold (reads≥3) were retained, followed by generating accurate and reliable clinical test reports automatically. Any pathogens identified in the NTC were disregarded, unless the reads per million (RPM) of the pathogen detected in the sample were ≥10 times that of the same pathogen found in the NTC.

### 2.8 Statistical analysis

The data were analyzed using SPSS 27.0 software (IBM SPSS, USA). For baseline characteristics, qualitative data were presented as numbers and percentages, while quantitative data were presented as mean ± standard deviation or as median and range (minimum and maximum). Comparative analyses were conducted using McNemar’s chi-squared test to evaluate the detection rates between mNGS and CT. P values < 0.05 were considered statistically significant.

## 3 Results

### 3.1 Patient and sample characteristics

In this study, a total of 85 kidney transplant recipients with infections were included, and their characteristics are presented in **Table 1**. Of the 85 patients, 40 (47.06%) were male and 45 (52.94%) were female, with an average age of 48.49 ± 10.34 years. The median time of infection after transplantation was 450.00 (range: 150.00 to1733.75) days.

**Table 1:** Baseline demographics and clinical characteristics of patients.

Characteristic	Value
Sex	
Male	40(47.06%)
Female	45(52.94%)
**Age, year**	48.49 ± 10.34
**Time after transplantation, day**	570.00(150.00, 1733.75)
Infection sites	
Lung infection	38(44.71%)
Fever	36(42.35%)
Urinary tract infection	11(12.94%)
Medication history	
No Antibiotic Used	6(7.06%)
Antibiotic Only	42(49.41%)
Antifungal Only	1(1.18%)
Antibiotic + Antifungal	14(16.47%)
Antibiotic + Antiviral	18(21.18%)
Antibiotic + Antifungal + Antiviral	4(4.71%)

### 3.2 Comparison of mNGS and CT in clinical diagnostic performance

**Figure 1:**
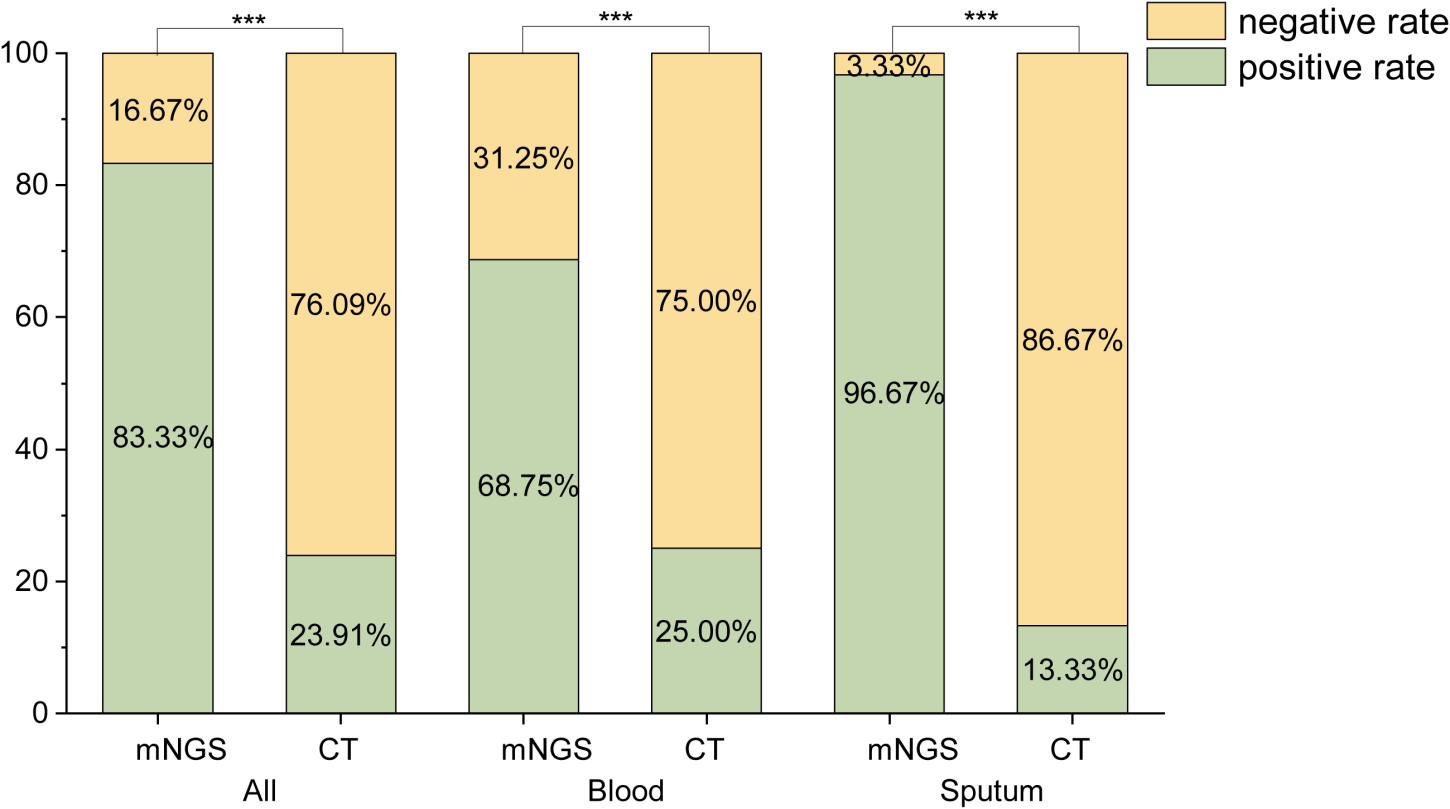
Comparison of the positive rates of mNGS and CT. ***:P<0.001.

The positive detection rates of mNGS and CT were compared, as shown in **Figure 1**. A total of 138 samples were analyzed, comprising 64 blood samples, 30 sputum samples, 22 BALF samples, 16 urine samples, 3 wound drainage fluid samples, 2 throat swabs, and 1 cerebrospinal fluid sample. Of these, 60 samples (43.48%) were sent for fever of unknown origin, 59 (42.75%) for pulmonary infection, and 19 (13.77%) for urinary tract infection. The positive rates of mNGS were significantly higher than those of CT (P< 0.001).

**Figure 2:**
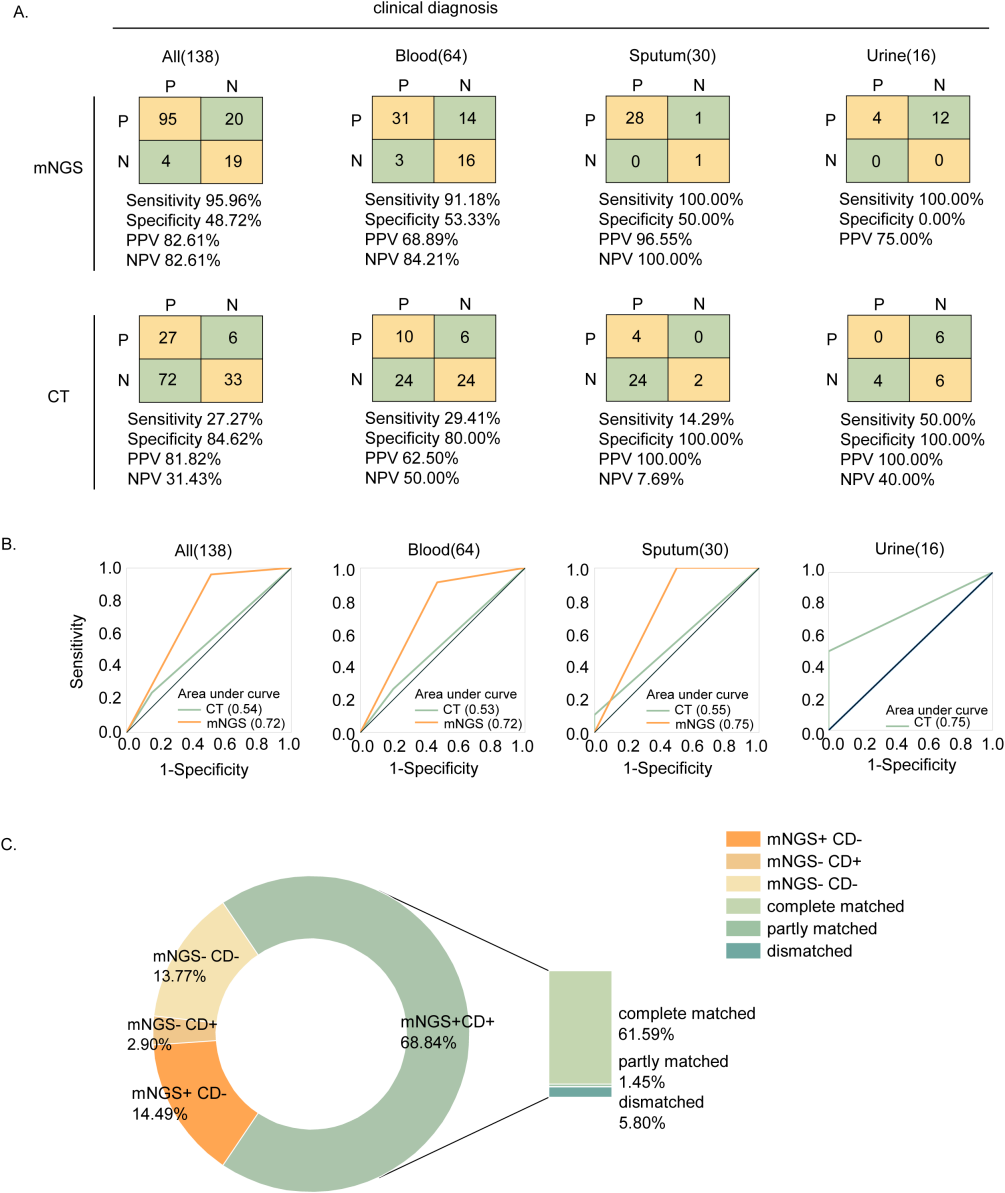
Performance and concordance analysis. **(A)** 2×2 contingency tables comparing the performance of mNGS and CT relative to clinical diagnosis for 138 samples. **(B)** Performance comparison of mNGS and CT using ROC curve. **(C)** Concordance of mNGS and clinical diagnosis.

We considered the clinical diagnosis as the gold standard. In comparison to this gold standard, the detection performance of mNGS and CT is shown in **Figures 2A, B**. Among the 138 samples, mNGS achieved a sensitivity of 95.96%, a specificity of 48.72%, a positive predictive value (PPV) of 82.61%, and a negative predictive value (NPV) of 82.61%. Compared to CT, mNGS exhibited higher sensitivity in all samples (95.96% vs. 27.27%), as well as in blood (91.18% vs. 29.41%), sputum (100.00% vs. 14.29%), and urine (100.00% vs.50.00%) samples. However, mNGS demonstrated lower specificity in all samples (48.72% vs. 84.62%), as well as in blood (53.33% vs. 80.00%), sputum (50.00% vs. 100.00%), and urine (0.00% vs. 100.00%) samples.

Among the 138 enrolled samples, 95 (68.84%) were detected as positive by both mNGS and clinical diagnosis. Of these, 85 (61.59%) cases were completely consistent, 2 (1.45%) cases were partially consistent, and 8 (5.80%) cases were completely inconsistent, as shown in **Figure 2C**. The partial consistency between mNGS and clinical diagnosis was mainly due to cases where patients were diagnosed with multiple pathogen infections, but mNGS detected only some of the pathogens and missed others. In 10 samples, mNGS failed to detect the presence of pathogens. The primary issues were that mNGS failed to detect severe acute respiratory syndrome coronavirus 2 (SARS-CoV-2) in 5 cases (50.00%) and *Candida albicans* in 1 sample (10.00%). Additionally, mNGS failed to identify any pathogens in 4 samples (3 blood and 1 cerebrospinal fluid), even though these patients were clinically considered infected, indicating the potential for missed detection using mNGS.

### 3.3 The spectrum of pathogens

**Figure 3:**
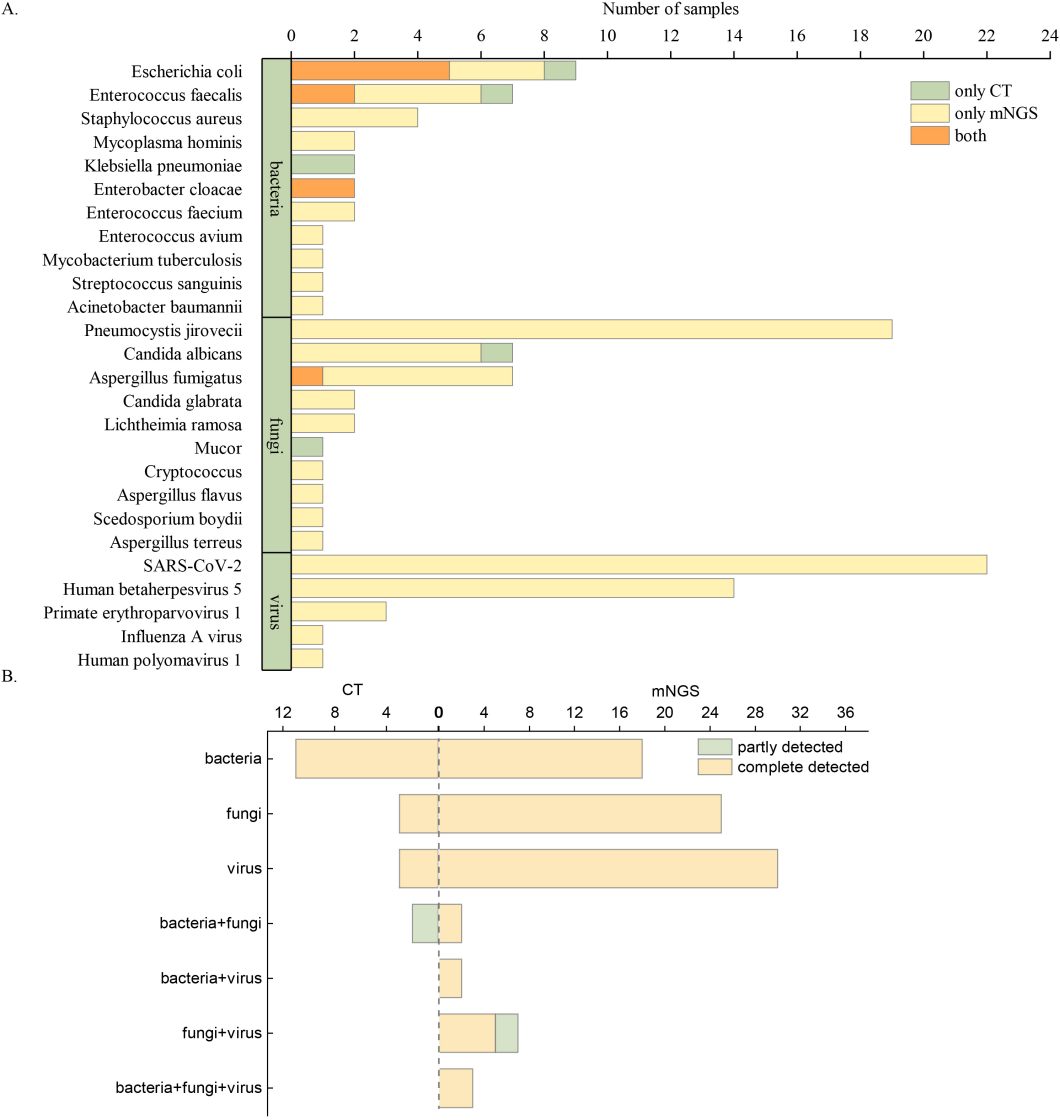
Pathogens detected by mNGS and CT. **(A)** Overview of clinically diagnosed pathogens detected by mNGS and CT. **(B)** Comparison of diagnostic performance between mNGS and CT in identifying single and multiple pathogens.

The clinically diagnosed pathogens detected by mNGS and CT are shown in **Figure 3A**. A total of 115 pathogens were detected in 138 samples. In kidney transplant patients, mNGS demonstrated a significantly higher positive detection rate (109, 94.78%) compared to CT (16, 13.19%, P < 0.01) for various types of pathogens. The top seven microorganisms detected in infected patients after kidney transplantation were SARS-CoV-2 (22, 19.13%), *Pneumocystis jirovecii* (19, 16.52%), CMV (14, 12.17%), *Escherichia coli* (9, 7.83%), *Enterococcus faecalis* (7, 6.09%), *Aspergillus fumigatus* (7, 6.09%), and *C. albicans* (7, 6.09%). Among the sample types analyzed by mNGS, sputum samples detected the most pathogens, with SARS-CoV-2 being the primary pathogen (16/35,45.71%). CMV (10/31, 32.26%), *E. coli* (6/17, 35.29%), and *P. jirovecii* (10/24, 41.67%) were the most common pathogens in blood, urine, and BALF, respectively, as shown in **Supplementary Figures 1**-**4**.

Notably, in our study, two recipients from the same donor developed a fever on the 7th day following kidney transplantation. *Lichtheimia ramosa*, a type of mucormycosis, was detected in the blood by mNGS. This finding alerted clinicians, prompting the rapid initiation of anti-infection treatment and a graft nephrectomy. The quick intervention ultimately benefited the patient’s survival. Subsequent culture tests, obtained at a later time, confirmed the diagnosis of mucormycosis.

In 12 patients, same pathogens were detected in paired sample types (e.g., blood and infection site samples) for each patient using mNGS, as shown in **Supplementary Figure 5**. This high concordance between blood and site of infection suggested that blood tests could serve as a viable alternative for pathogen detection in these patients. Moreover, the relative abundance of the pathogen tended to be higher in blood compared to the paired sample type, suggesting that certain pathogens may be easier to detect in blood.

The diagnostic capabilities of mNGS and CT for single and multiple pathogen infections are shown in **Figure 3B**. Overall, CT results in kidney transplant patients were predominantly negative. Even when CT results were positive, they usually identified only a single pathogen, primarily bacteria or fungi. In contrast, mNGS demonstrated remarkable detection performance, identifying not only single pathogen but also multiple pathogens, involving bacteria, fungi, and viruses.

### 3.4 Significance of mNGS in clinical treatment decision-making

**Figure 4:**
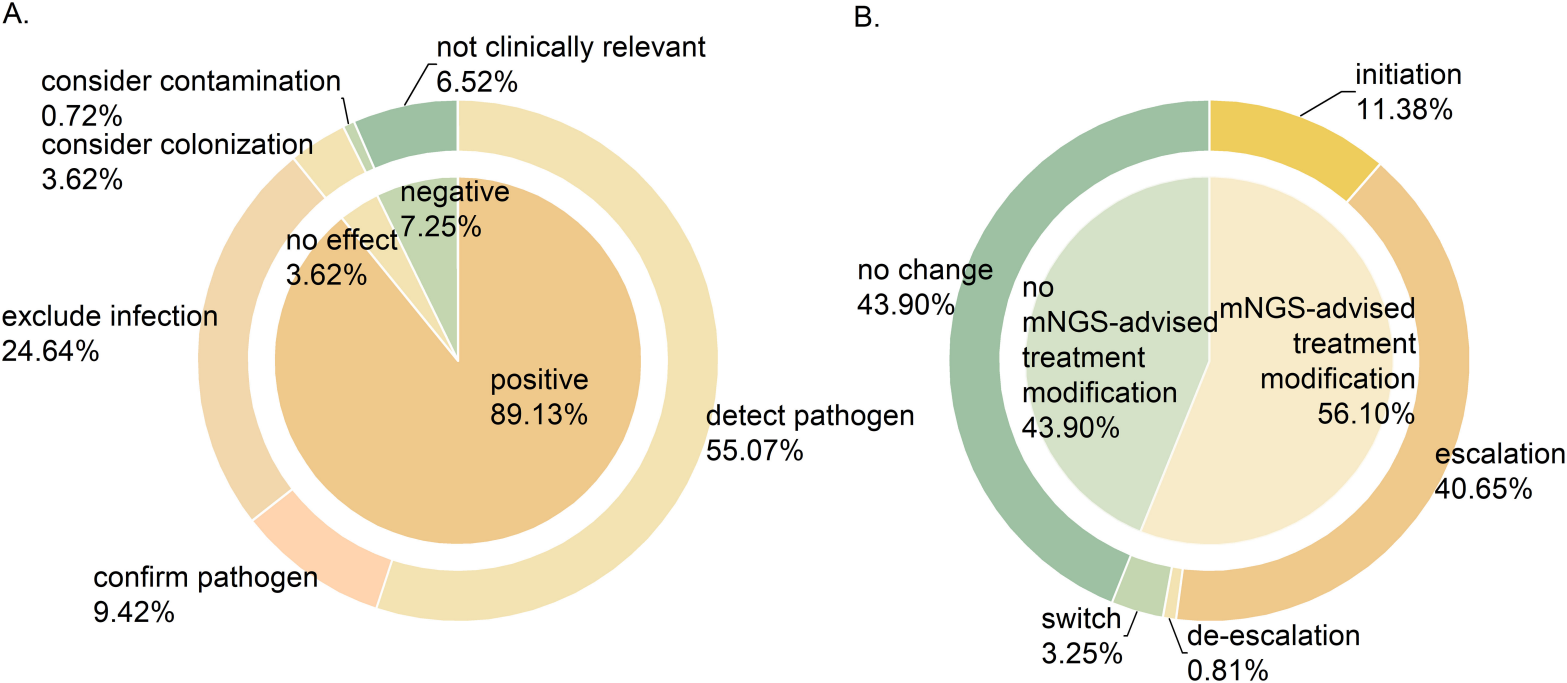
The significance of mNGS in clinical application. **(A)** The clinical value of mNGS. **(B)** The impact of mNGS results on medication regimens.

Clinical experts assessed the clinical value of mNGS results of 138 samples, as shown in **Figure 4A**. Of these, 123 samples (89.13%) had a positive impact on clinical diagnosis and treatment, encompassing pathogen identification (76, 55.07%), exclusion of infection (34, 24.64%), and pathogen confirmation (13, 9.42%). Additionally, mNGS results had no impact on 5 (3.62%) samples, mainly because the detected species were considered colonizing bacteria. The impact of mNGS results was negative on 10 (7.24%) samples, either due to contamination (1, 0.72%) or because the findings were irrelevant to clinical symptoms (9, 6.52%).

The impact of mNGS results on clinical medication plans is shown in **Figure 4B**. Among 123 samples where mNGS showed positive clinical value, the results prompted treatment modification in 69 (56.10%) patients, with 50 (40.65%) receiving escalated treatment plans. This escalation included upgraded antibiotic regimens and combination therapies involving antibacterial, antifungal or antiviral medications. The treatment regimens were initiated in 14 (11.83%) patients, de-escalated in 1 (0.81%), and switched in 4 (3.25%). In addition, the treatment regimen remained unchanged in 54 patients (43.90%).

By performing mNGS on six patients at multiple time points after transplantation, we observed a decrease in relative abundance of pathogens in five patients who had their medication adjusted based on the mNGS results, as shown in **Supplementary Figure 6**. Conversely, only one patient showed an increase in relative abundance. In Patient 16 (P16), the initial detection of *E. faecalis* showed low relative abundance, which did not prompt clinical attention or a targeted change in the treatment plan according to the mNGS results. However, a subsequent adjustment to the treatment plan based on a second mNGS test led to improved clinical symptoms and a positive prognosis for this patient.

## 4 Discussion

The primary objective of this study was to assess the clinical utility of mNGS in the diagnosis and treatment of kidney transplant patients. We found that mNGS demonstrated higher positive rates and superior sensitivity but lower specificity in different sample types compared to CT, consistent with findings from previous studies (Miao et al., 2018; Liu et al., 2023; Pang et al., 2023; Zhu et al., 2023; Chen et al., 2024; Lv et al., 2024). Notably, the sensitivity of CT in our study was lower than previously reported. This may be attributed to the fact that 92.94% of the kidney transplant patients in our study were receiving antimicrobial treatment upon admission. Unlike CT, mNGS was less influenced by prior antibiotic use, so its sensitivity and specificity aligned with previously reported data (Miao et al., 2018; Hao et al., 2023; Nie et al., 2023).

While mNGS offers unparalleled sensitivity, it also presents challenges in pathogen detection. First, mNGS can identity pathogens at minimal levels, which are often considered clinically irrelevant. Second, viral dynamics in immunocompromised individuals are complex (Funk et al., 2007), making it difficult to distinguish between latent viruses and active infections in these patients. Moreover, when mNGS is applied to asymptomatic patients, results can be significantly influenced by commensal microbes, further complicating the interpretation of the findings. The high sensitivity might lead to difficulties in interpretation, overdiagnosis, and complications in clinical management. Therefore, it is essential to remember that mNGS is merely an auxiliary diagnostic tool, and its results should be interpreted in conjunction with the patient’s clinical symptoms.

The lower specificity of mNGS, particularly in blood samples, can be attributed to several factors. Due to its high sensitivity and unbiased nature, mNGS is susceptible to contamination from environmental DNA, which may be mistakenly identified as pathogens (Han et al., 2019). For example, during blood samples collection, microbial contamination from the surface of the skin may compromise the specificity of mNGS (Liumbruno et al., 2009). Furthermore, viruses are the frequently detected in blood mNGS testing, but the presence of these viruses can indicate various conditions, such as latent infections, reactivations, or active infections (Park et al., 2023). Misclassification of latent viruses as pathogenic organisms leads to reduced specificity. These false-positive results may significantly impact clinical decision-making, potentially prompting unnecessary treatments and wasting resources. To enhance the specificity of mNGS, several strategies have been implemented, including the use of advanced bioinformatics for quality read filtering and contaminant removal, distinguishing true positives from background noise, incorporating negative controls to address contamination, creating a background microbial database to filter out environmental contaminants, and adjusting read cutoffs to minimize false positives from low-confidence sequences. Most importantly, in clinical practice, it is essential to complement mNGS results with clinical context and other diagnostic methods to improve the reliability and accuracy of the findings.

Our study demonstrated that mNGS provided distinct advantages in detecting multiple pathogens in kidney transplant patients with infections. Notably, the most frequently observed co-infections involved fungal and viral pathogens. Generally, the immunocompromised patients had a higher prevalence of co-infections compared to those without immunodeficiency, with bacterial-fungal-viral infections being the most common (Wu et al., 2020; Li et al., 2022). These findings might be attributed to the weakened immune systems of kidney transplant recipients, rendering them more susceptible to both common and opportunistic infections.

By analyzing the pathogen spectrum, we found that the most frequently detected pathogens in kidney transplant patients in our study were *P. jirovecii*, CMV and SARS-CoV-2. In line with previous studies, mNGS exhibited higher sensitivity than conventional techniques in detecting *P. jirovecii*, particularly in BALF samples from kidney transplant recipients (Wang et al., 2019; Irinyi et al., 2020; Li et al., 2020; Xie et al., 2021; Zhong et al., 2023). In previous studies, the rates of infection with *P. jirovecii* after renal transplantation were reported to be 23.21-28.14%, while in this study, the rate was 16.47% (Yang et al., 2021; Chen et al., 2022). This lower rate may be primarily due to our adherence to the guidelines recommending the use of trimethoprim-sulfamethoxazole (TMP-SMX) for infection prevention during the first 3 to 6 months after kidney transplantation. In fact, among the 14 patients who were infected, only 2 developed infections within 5 months after transplantation, while the remaining patients developed infections 6 months or more after transplantation (Cheng et al., 2024). The diagnosis of *P. jirovecii* pneumonia in these patients was confirmed after they presented with symptoms, with subsequent chest computed tomography scans revealing abnormalities, and mNGS results providing definitive evidence. The integration of these diagnostic modalities was imperative for establishing a definitive diagnosis of PJP, as the non-specific nature of chest computed tomography scans and serum BDG tests (Alanio et al., 2016; White et al., 2017). In patients with positive mNGS results for PJP, the computed tomography imaging all suggested pneumonia, and some patients (35.71%) had positive BDG results in blood samples collected at the time of admission.

Additionally, the high detection rate of human herpesviruses, especially CMV, might partially reflect the weakened immune system of kidney transplant patients (Abou-Jaoude et al., 2021). CMV infection can lead to decreased survival rates for transplant recipients and increased risks of rejection; therefore, accurate monitoring of CMV infection during clinical diagnosis and treatment is meaningful (De Keyzer et al., 2011; Dobrer et al., 2023; Manuel et al., 2024). The high detection rate of SARS-CoV-2 in this study was likely due to the timing of sample collection during the SARS-CoV-2 pandemic. We found that SARS-CoV-2 was predominantly detected in sputum and less frequently in BALF. This may be explained by the initial invasion of SARS-CoV-2 in the upper respiratory tract, specifically targeting nasopharyngeal and/or oropharyngeal tissues (Harrison et al., 2020; Hui et al., 2020; Cevik et al., 2021). This may be due to the high affinity between SARS-CoV-2 and angiotensin-converting enzyme 2 (ACE2) (Hamming et al., 2004; Ziegler et al., 2020). In our study, the detection was conducted during the nascent period of infection, suggesting that sputum mNGS could serve as an effective method for early diagnosis of upper respiratory tract viral infection, such as SARS-CoV-2, in kidney transplant patients. The most prevalent pathogens in patients with urinary tract infections after kidney transplantation were *E. coli*, *E. faecalis* or *E. faecium*, and *E. cloacae*. However, when considering factors such as detection rate, detection time, and economic benefits, mNGS did not appear to offer apparent advantages over CT in diagnosing urinary tract infections.

In the realm of clinical practice, the pre-emptive diagnosis of rare and clinically elusive species poses a significant challenge. In this study, two patients were diagnosed with mucor infection after kidney transplantation, which was a rare but often fatal infection in transplant recipients (Song et al., 2017; Zhang et al., 2022; Miller et al., 2021). The diagnosis and treatment process of these two patients underscored the pivotal role of mNGS in the early detection of such infections. The rapid turnaround time of mNGS results was instrumental in prompting swift clinical responses and enhancing survival rates for kidney transplant recipients.

Moreover, this study assessed the clinical utility of mNGS in the diagnosis and treatment of kidney transplant recipients. mNGS demonstrated a positive impact in 89.13% of cases, primarily by facilitating pathogen detection (55.07%). It was noteworthy that 43.90% of mNGS results did not prompt alterations in clinical treatment plans, partially because the prior empirical medications used upon admission had already targeted the detected pathogens.

Lastly, it is valuable to perform mNGS tests at multiple time points after solid organ transplantation to monitor the infection status. After adjusting the treatment plans based on the mNGS results, four patients showed a decreasing trend in normalized reads of pathogens, which promoted favorable outcomes. Only two patients exhibited a continued increase in pathogen reads. One of them did not adjust the treatment plan based on mNGS, and the other had multiple underlying diseases, resulting in a poor prognosis. By analyzing the variations in reads, clinicians can obtain critical insights into the patient’s condition, potentially leading to more personalized and timely interventions.

mNGS is a powerful tool for pathogen detection, but its utility can be diminished in certain scenarios. For example, in non-sterile samples like BALF and sputum, the detection of both pathogenic and non-pathogenic microorganisms can lead to confusion due to excessive data on harmless microbes. Additionally, when traditional diagnostic methods such as PCR, culture, or antigen tests are well-established and sufficiently sensitive, mNGS may not provide added value, as conventional tests are typically cheaper and easier to interpret. Moreover, the high costs associated with mNGS assays—ranging from $1,000 to $2,500—along with the need for specialized equipment and expertise may further hinder its adoption in resource-limited settings (Chiu and Miller, 2019; Ramachandran and Wilson, 2020; Li et al., 2021; Diao et al., 2022). Overall, while mNGS excels in certain contexts, its utility decreases when traditional methods are effective, timely, and cost-efficient.

This study had certain limitations. First, a subset of samples was not subjected to real-time PCR analysis. Second, the sample pool was derived from a single-center retrospective study, highlighting the need for prospective and multicenter studies for validation. Third, the study included a relatively narrow range of sample types, neglecting other samples such as tissues and cerebrospinal fluid, which also hold significant clinical value. Fourth, the lack of specific clinical data, such as hospitalization duration, mortality rates, and overall costs, hindered the discussion of the role of mNGS in improving patient prognosis and alleviating healthcare burdens. Lastly, interpretation of mNGS reports needed to be standardized for enhanced accuracy and reliability.

In conclusion, we found that mNGS was highly effective in detecting respiratory infections after kidney transplantation, particularly those caused by specific pathogens such as *Pneumocystis jirovecii*, *Aspergillus*, and rare infections caused by mucor. mNGS accelerates diagnosis and guides treatment decisions while playing a crucial role in monitoring treatment efficacy, which allows for real-time adjustments to treatment plans. However, it is not recommended as a primary approach for diagnosing urinary tract infections. Overall, mNGS enhances clinical diagnosis and treatment, leading to better patient outcomes. Therefore, it’s crucial to consider mNGS as a valuable adjunctive test for detecting pathogens after kidney transplantation.

## Data Availability

The original contributions presented in the study are included in the article/**Supplementary Material**. Further inquiries can be directed to the corresponding author. The datasets generated for this study can be accessed through the GSA database under the bioproject accession number PRJCA032130.
