# Supplementary figures and images for: Deciphering the pathogen heterogeneity for precise diagnosis and personalized therapeutics of infections after kidney transplantation: insights from metagenomic next-generation sequencing
Source: Front Cell Infect Microbiol. 2024 Nov 14;14:1456407. doi: 10.3389/fcimb.2024.1456407 (PMC11602478; doi:10.3389/fcimb.2024.1456407)

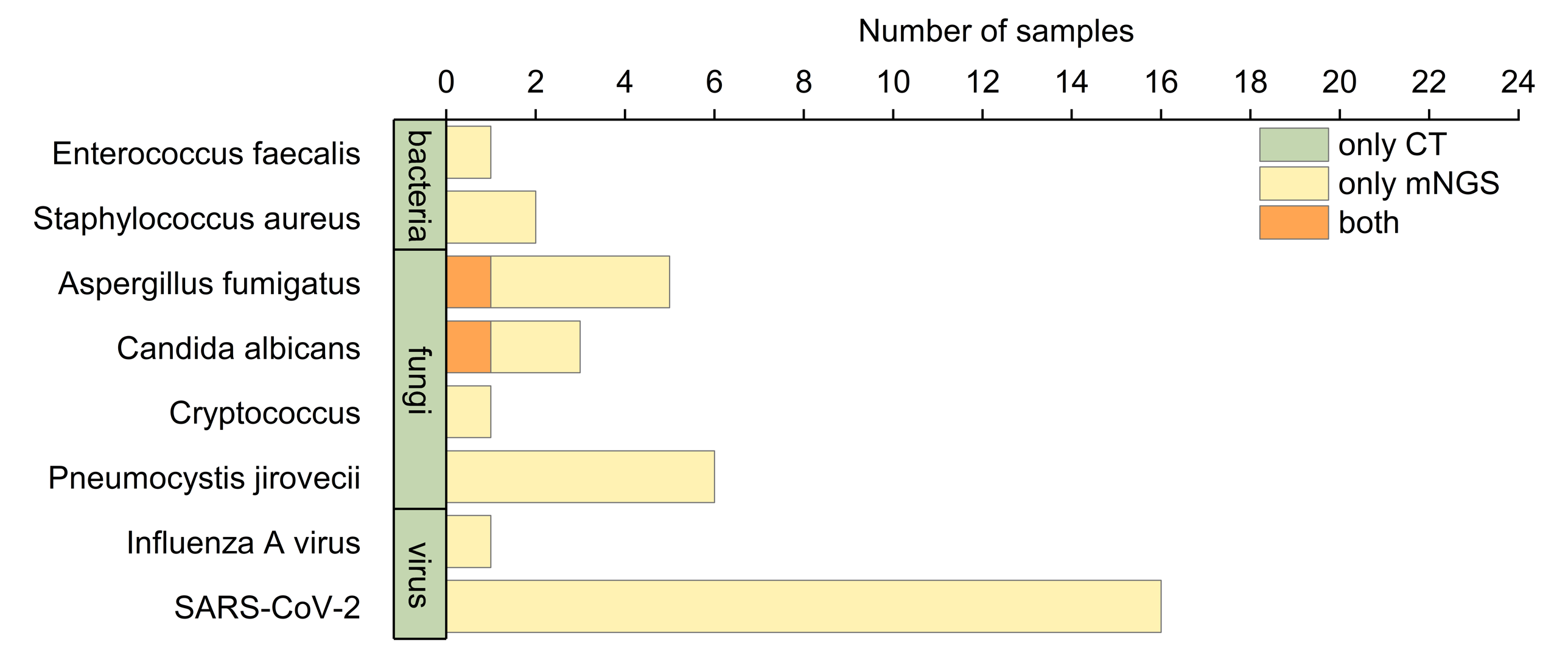

Supplement: Supplementary file 2 [file Image2.jpeg]

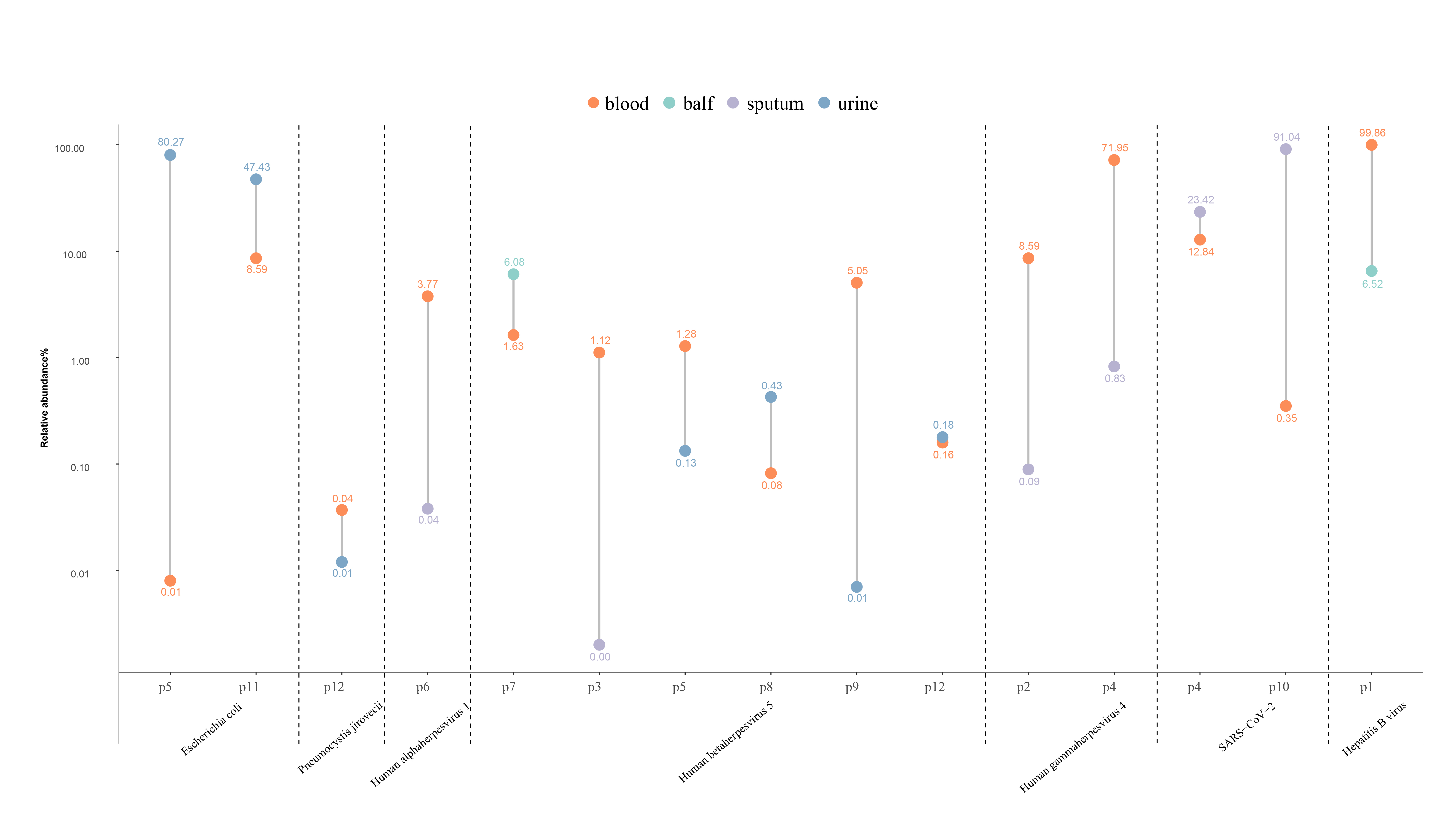

Supplement: Supplementary file 5 [file Image5.jpeg]

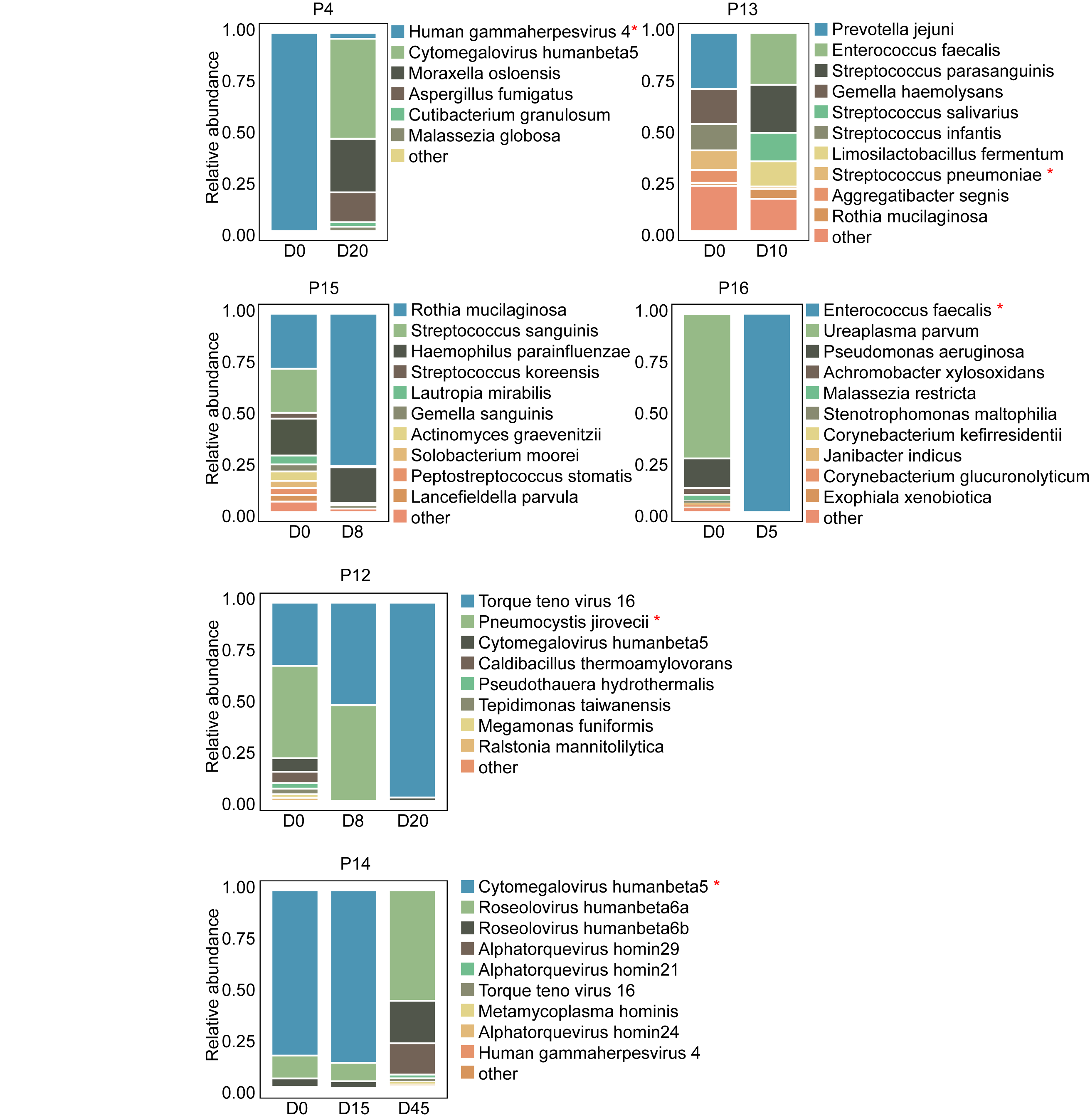

Supplement: Supplementary file 6 [file Image6.tif]
